# Longitudinal Effects of Mediums of Word Explanation on L2 Vocabulary Learning Strategies Among Chinese Grade-7 Students

**DOI:** 10.3389/fpsyg.2020.00702

**Published:** 2020-04-24

**Authors:** Yang Dong, Yi Tang, Sammy Xiao-Ying Wu, Wei-Yang Dong, Zhen Li

**Affiliations:** ^1^Department of Social and Behavioural Sciences, City University of Hong Kong, Kowloon, Hong Kong; ^2^School of Economics and Management, China University of Petroleum, Qingdao, China; ^3^Department of Special Education and Counselling, The Education University of Hong Kong, Tai Po, Hong Kong; ^4^Department of Asian Policy Studies, The Education University of Hong Kong, Tai Po, Hong Kong; ^5^Department of Chinese Language Studies, The Education University of Hong Kong, Tai Po, Hong Kong

**Keywords:** strategy use, mediums of word explanation, second language acquisition, intervention, vocabulary

## Abstract

This longitudinal study investigated how different mediums of word explanation affected the use of English vocabulary strategies among Chinese Grade-7 students. 170 students were tested on their English receptive vocabulary size and vocabulary strategy application before and after an 8.33-month intervention. Students were divided into three experimental groups and one control group. The three experimental groups were provided with learning materials that explained the target vocabulary in three mediums, respectively: English-only, English-and-Chinese, and Chinese-only. Results showed that, after the intervention, receptive vocabulary size did not have any direct significant impact on vocabulary strategy development, whereas mediums of word explanation materials impacted students’ application of vocabulary learning strategies (VLS) in different ways. Our findings showed that the English-only mediums significantly enhanced students’ use of metacognition, cognition, and memorization strategies, but decreased social strategy development. Chinese-only mediums significantly facilitated cognition and memorization strategy development. Implications for L2 vocabulary education are discussed.

## Introduction

Many researchers have investigated how to develop readers’ vocabulary learning strategies (VLS) effectively (e.g., Erman et al., 2016; Vedder and Benigno, 2016; Caro and Mendinueta, 2017; Sheridan and Markslag, 2017; Ahmad et al., 2018), including lexical inferring (Hamada, 2015; Shahar-Yames and Prior, 2018) and memorization strategies (Wahlheim et al., 2016; Wang and Kelly, 2017). Researchers put forward a hypothesis that the appropriate medium of word explanation ought to promote the development of VLS under second language (L2) acquisition (e.g., Nosratinia and Zaker, 2015; Hwang and Wang, 2016; Sheridan and Markslag, 2017); however, very few studies have touched on this field. Besides, even fewer studies have investigated word explanation under sentence level. Enlightened by Schmitt’s (1997) taxonomy of VLS, this study examines whether different mediums of word explanation, namely, explanatory materials presented in L1, or L2, or a combination of both, would affect learners’ cognitive processing of metacognitive, cognitive, memorization, and social strategies.

## Literature Review

### The Classification of Vocabulary Learning Strategies

Vocabulary learning strategies pertain to actions that a learner takes to facilitate the completion of learning vocabularies (Gu, 2018). Empirical studies showed that effective VLS would enhance L2 language proficiency (Ajayi, 2015; Zhang and Lu, 2015; Susanto and binti Ab Halim, 2017), such as enlarging vocabulary size (e.g., Hamzah et al., 2009), and further determined second language context comprehension (Vedder and Benigno, 2016; Caro and Mendinueta, 2017). Understanding what kinds of VLS students adopt, and how students adopt them, is helpful for L2 educators to assist learners in internalizing meanings of target vocabulary, particularly receptive vocabulary knowledge (Wang, 2015; Rose et al., 2018).

Schmitt (1997) developed a comprehensive inventory for VLS, which involves two dimensions: discovery and consolidation. Discovery strategies are used when “learners are faced with discovering a new word’s meaning without recourse to another person’s experience” (Schmitt, 1997, p. 205). This strategy is unrepresentative as it might not be frequently used if the purpose is to explicitly memorize the newly learned target words (Nassaji, 2003). The consolidation dimension refers to learners consolidating the meanings of new words when they encounter them again, which highlighted the effect of the target word’s semantic meaning recognition. In the consolidation dimension, strategies were classified into five types: meta-cognitive, cognitive, memorizing, determination, and social strategies (Schmitt, 2000). The meta-cognitive strategy refers to having a conscious mind of the learning process and being able to plan, monitor, or even evaluate the learning progress. The memorization strategy, known as mnemonics, is about associating new words with previously learned knowledge, such as using forms of imagery and grouping. The cognitive strategy does not involve manipulative mental processing but includes repetitive and mechanical methods to learn vocabulary, such as using word lists, flashcards, and taking notes. The determination strategy refers to the learner’s ability to select the appropriate resources to know the target word meaning. The social strategy involves asking for help or collaborative study and adopts in both discovery and consolidation dimensions as it could be used for both purposes. In the current study, we investigated the effects of mediums of word explanation on the meta-cognitive strategy, cognitive strategy, memorizing strategy, and social strategy. We have removed the determination strategy because the learning materials of the current study were limited, and students were provided with specific learning materials.

### Past Studies on VLS Development

Following Krashen’s (1989) input-oriented language acquisition theory, researchers found that engaging L2 language learners in extensive reading could develop their VLS, which may facilitate their L2 vocabulary learning (e.g., Nassaji, 2003; Webb, 2008; Zhang and Lu, 2015). That is, when learners are exposed to meaningful L2 inputs, they are usually encouraged to assess word meanings by employing various strategies such as meta-cognition, memorization, and cognition strategies (Ho et al., 2016; Yeldham, 2016; Barnes and Dickinson, 2017). Past studies revealed that L2 learners exposed to authentic reading materials experience significant progress in VLS use (Sun et al., 2016; Arozaq et al., 2017; Schwartz et al., 2017). For instance, when they encountered new words while reading an article written in the target language, students would adopt a series of strategies such as looking up new words in dictionaries, consulting their teachers, and guessing the meanings of new words (e.g., Currie and Cain, 2015; Koyama, 2015; Graves et al., 2018). Gu (2018) used think-aloud protocols and interviews in her study of Chinese university learners of EFL and found that the students adopted meta-cognitive strategies, such as meta-jargon explanation, to figure out the meanings of new words when reading authentic texts.

Word incidental learning was another effective way to promote students’ VLS development (Cooper, 1999; Baleghizadeh and Shahry, 2011). Word incidental learning is a type of functional input via which learners could make informed guesses about the meaning of a new word. Past studies show that reading texts through word incidental learning determined the ways in which readers interpret the meaning and the key term or words explanation, which supports the main idea that sentences increase the global inference (Pam and Karimi, 2016; Lee, 2017). Oxford (2002) and Friend et al. (2018) reported that compound words or phrases contributed to memorization strategy development through semantic association. However, past studies investigated the effect of word incidental learning on students’ VLS development under the level of compound words or phrases, and a few studies examined the word incidental learning effect on VLS development through a sentence-level explanation. Moreover, the interaction between the input-oriented language acquisition and the semantic recognition effect of consolidation was unknown.

### VLS Examination

In most studies, VLS was examined in reading comprehension activities. An efficient reader could make use of the available reading context or available cues to enhance reading performance. For example, students may intentionally skip unfamiliar words in a reading task (Ardasheva et al., 2017; Suk, 2017; Wright and Cervetti, 2017). In this case, students may not even use any VLS to learn unfamiliar words, as a lower percentage of unfamiliar words does not present an obstacle to reading-materials comprehension. To avoid students’ neglect of digesting unfamiliar words when reading L2 texts, this study attempts to use single sentence reading materials in response to single strange word explanations, which means that target words are explicitly presented to students with available linguistic cues to support their inference-making or understanding. Besides, past studies on VLS mainly focused on L1 VLS. Little is known about the specific effects of different mediums of word explanation on learners’ L2 VLS, including metacognitive, cognitive, memorization, and social strategies. Even less is known about the interaction effect of mediums of word explanation on semantic recognition.

### Chinese Script

Logographic and alphabetic scripts have different characteristics, including morphologies, mappings among orthography, semantic rules, and phonology (Tsai et al., 2012; Ehrich et al., 2013). Specifically, Chinese characters are the basic writing units, constituted by strokes (Anderson et al., 2013; Chen et al., 2013; Chang et al., 2014). Many Chinese characters have a phonetic radical and a semantic radical. Phonetic radicals provide hints on the character pronunciation. Semantic radicals carry information about character meaning, which determines the interpretation of the reading comprehension of the readers. The print-sound mappings are ambiguous in Chinese. Past studies have shown that phonological skills in Chinese had a lower correlation with reading comprehension than in alphabetic scripts (Gottardo et al., 2001). The different effect of metalinguistic knowledge may impact the development of VLS through different mediums of word explanation. A few studies have investigated the effects of mediums of word explanation on VLS development in Chinese students. Moreover, the effect of input theory on consolidation and semantic recognition under logographic scripts has been investigated even less with learners undertaking L2 vocabulary knowledge acquisition.

### The Current Study

This study examines the potential effects of different word learning contexts on VLS among Chinese EFL students. Specifically, the current study used three kinds of mediums of word explanation as vocabulary learning materials to test their VLS use: L1 explanation, L1-and-L2 explanation, L2 explanation. The correlation between VLS and different explanatory mediums was later examined.

## Materials and Methods

### Participants

Participants were included in the study following the completion of an informed consent form from both parents and students. The current study selected 170 Grade-7 Chinese EFL students (74 boys and 96 girls) from four classes in a government-funded secondary school in Shenzhen, which is a major manufacturing city in South China. The average age was 12.04 years old (*SD* = 0.68). All 170 students came from a low-income family whose household income was below 25% of the household income in the city of Shenzhen. Specifically, Class 1 had 42 students (21 boys and 21 girls, mean age = 11.98 years, *SD* = 0.68), Class 2 had 44 students (16 boys and 28 girls, mean age = 12.25 years, *SD* = 0.69), Class 3 had 42 students (20 boys and 22 girls, mean age = 11.93 years, *SD* = 0.68), and Class 4 had 42 students (17 boys and 25 girls, mean age = 12.00 years, *SD* = 0.66). All selected students had a similar academic performance before the study commenced, based on the school database record. Besides, all participants were taught by one English teacher in the 2016–2017 academic school year.

In China, students begin to undertake formal and systematic English lessons from grade 7, which is the first year of secondary school. In primary schools, students gain a basic knowledge of English at the conversational level, such as daily greetings. The grammatical knowledge, fruitful literacy knowledge, and English learning strategy are not being taught to students systematically in school. The teaching focus is on developing students’ basic communication skills and daily conversations in English, with an emphasis on enjoyable classroom activities, such as singing and playing (Gill et al., 2008). The graduation requirement of the English receptive vocabulary size was 580, at which point students could understand a basic sentence in the Longman English Dictionary (Horst and Collins, 2006).

### Measurements

#### Receptive Vocabulary Size Test

We adopted the Receptive Vocabulary Size Test from Liao’s (2012) receptive vocabulary test, in which all tests consisted of three levels, which were selected from three 1000-frequency vocabulary lists. Each level had 10 words, for example, level 1 had 10 words to represent the first top 1000 frequency words from the total vocabulary list. Liao’s test was revised from the Nation’s (2008) standardized scale of receptive vocabulary measurement. This receptive vocabulary measurement showed a high validity among Chinese participants in secondary school. The first part represented the most frequently used 1000 words, while the second part tested the receptive vocabulary from the frequency list from 1001 to 2000. The third part examined the receptive vocabulary from the frequency list from 2001 to 3000. The frequency of word use decreased gradually from part one to part three of the tests. Only when the participant had answered at least nine questions correctly in one 1000-word level test could he or she move to the next level. One correct answer was awarded 1 point, and the maximum score for this test was 30. Cronbach’s alpha internal consistency reliability for the current study was 0.92. An example test item is the following:

SEE: They saw it.
a. cut b. waited for c. looked at d. started.

#### Vocabulary Learning Strategies

The VLS scale was a researcher-developed VLS scale by Zheng (2015), which was developed from Schmitt’s (1997) VLS scale. With the exception of the determination scale, the whole VLS scale contains 39 items, ranking on a five-point Likert scale from “Never or almost not true to me” (one count) to “Always or almost true to me” (five counts). We removed the determination strategy part because the current research focus was on examining the potential effects of vocabulary contexts, and students were required to use the provided materials only to learn vocabulary. Therefore, the determination strategy was ruled out to eliminate the potential effect of interfering with the results as students may use it to check reference materials, such as a dictionary. This study selected meta-cognition, cognition, social, and memorization strategies to examine the effects of vocabulary contexts on VLS. The Cronbach’s alpha internal consistency reliability for this sample was 0.83.

#### Academic Scores

The current study got selected students’ academic performance in Chinese, Math, English, Art, and PE from the school database. The last scores came from the entrance exam.

### Learning Materials

This study selected 2500 words from the China English Curriculum (2015) syllabus (Ministry of Education, 2003; Wang and Treffers-Daller, 2017), which was a high-frequency vocabulary list in English (Gyllstad et al., 2015; Tan et al., 2016). The explanation scripts (sentence level) came from two dictionaries: an English script from the Longman Dictionary and Chinese scripts from Hanyu Da Cidian (Chinese Dictionary). The current study further provided three versions of explanation on the selected English words. Version I referred to English words with an English explanation script only (EE); Version II, English words with both Chinese and English explanation scripts (ECE); Version III, English words with a Chinese explanation script only (EC). Take the target word “mountain” as an example:xf

Version I (EE). Mountain: “a very high hill.”
Version II (ECE). Mountain: “a very high hill.” “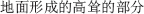.”
Version III (EC). Mountain: “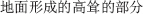.”

### Research Design

#### Data Collection

Participants were included in the study following the completion of an informed consent form from both parents and students. The current study followed a pre-test and a post-test research design, whereby all selected students were required to undertake a receptive vocabulary size test and VLS twice. Time 1: At the third week of September, all selected students were required to undertake a receptive vocabulary test and VLS. It took about 40 min under the supervision of one trained research assistant. Time 2: A post-test was carried out 2 weeks after all 8.33 months’ intervention. All selected students were required to undertake a receptive vocabulary size test and VLS. It took about 40 min under the supervision of one trained research assistant.

#### Learning Procedure

Version I to III had been assigned to three different classes. After random selection, Class 1 was required to use Version I, Class 2 was required to use version 2, and Class 3 was required to use Version III. Class 4 was not provided the same vocabulary list every day, without any explanation on each of the target words during this learning period. Class 1, Class 2, and Class 3 were experimental groups, while Class 4 was the control group. The only difference of materials application for the students of the four classes was that the medium of word explanation for each class was different, while the other English learning materials (learning target vocabulary list) were all the same every day.

Before the intervention commenced, the instructions and requirements were introduced through the English learning materials (Version I to III) to Class 1, Class 2, and Class 3 students (10 min). Class 4 was not provided any instruction related to materials use in order to control the effect of the medium of word explanation on VLS development.

#### Intervention Procedure

The English word learning materials needed around 10 min to finish every day. The experimental group students (Class 1–3) were required to receive the given message, which was sent out via a WeChat (an app for communication online) group every day during the intervention, following which they were required to copy the given words with the given script explanation, respectively. The learning English words list was the same for all experimental group students, with 10 new words each day. The experimental group students were required to write down the given words with explanation in the English exercise book as daily homework. The homework was checked by a trained research assistant in the next school days. The control group students received the same English words list from WeChat, except without any instruction.

**FIGURE 1:**
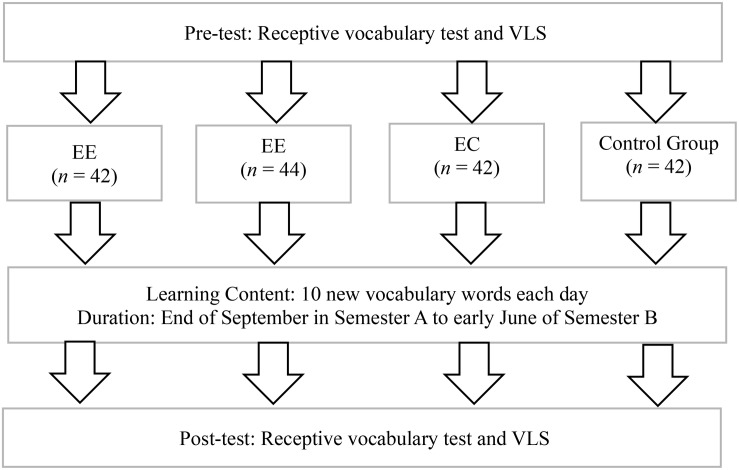
Research design.

All selected students (170 students) were taught the same content during daily school English courses by one English teacher. The English teacher was required to not mention any information of the instruction about the medium of word explanation script use before the intervention program commenced (see Figure 1).

#### Duration

The intervention program started at the end of September and ended in early June. The total intervention lasted around 8.33 months with an average learning time (learning materials exposure) of 41.67 h for each experimental group (Class 1–3) student.

### Data Analysis

The current study used R to conduct data analysis. A two-way ANOVA and a multiple Wilcox test were used for three mediums of word explanation examinations.

## Results

The results are presented in four parts. The first part shows a descriptive analysis of receptive vocabulary size and statistical effects of the four VLS. The second part used a two-way ANOVA to examine the four classes of students’ performance in receptive vocabulary size and VLS. Third, a multiple Wilcox test was used for difference comparison between the pre-test and post-test. Lastly, three dummy groups were created and assigned the control group as the basic-line group, to examine the effect of vocabulary size on VLS development by controlling for the medium of word explanation.

### Descriptive Analysis

Table 1 presents all four classes of students’ performance in vocabulary size and VLS in pre- and post-tests. At pre-test, Class 1 (EE) students scored 5.95 (*SD* = 0.82) in receptive vocabulary size test, the meta-cognition score was 3.12 (*SD* = 0.77), the cognition score was 2.17 (*SD* = 0.62), the memorization score was 3.86 (*SD* = 0.35), and the social score was 2.74 (*SD* = 0.54). Class 2 (ECE) students scored 5.91 (*SD* = 0.77) in receptive vocabulary size test, the meta-cognition score was 3.34 (*SD* = 0.78), the cognition score was 2.05 (*SD* = 0.57), the memorization score was 3.77 (*SD* = 0.52), and the social score was 2.68 (*SD* = 0.47). Class 3 (EC) students scored 5.83 (*SD* = 0.79) in receptive vocabulary size test, the meta-cognition score was 3.38 (*SD* = 0.85), the cognition score was 2.10 (*SD* = 0.58), the memorization score was 3.98 (*SD* = 0.41), and the social score was 2.71 (*SD* = 0.51). Class 4 (control group) students scored 5.93 (*SD* = 0.81) in receptive vocabulary size test, the meta-cognition score was 3.33 (*SD* = 0.75), the cognition score was 1.91 (*SD* = 0.66), the memorization score was 3.93 (*SD* = 0.46), and the social score was 2.67 (*SD* = 0.48). All skewness and kurtosis indicators were within ± 3, which showed that the scores were in a normal distribution at pre-test.

**TABLE 1:** Descriptive analysis of vocabulary size and vocabulary learning strategies in pre-test and post-test results.

		EE (*n* = 42)		ECE (*n* = 44)		EC (*n* = 42)		Control (*n* = 42)		*F*
		Mean	SD	Mean	SD	Mean	SD	Mean	SD	
Pre-test	Vocabulary size	5.95	0.82	5.91	0.77	5.83	0.79	5.93	0.81	0.17
	Metacognition	3.12	0.77	3.34	0.78	3.38	0.85	3.33	0.75	0.94
	Cognition	2.17	0.62	2.05	0.57	2.10	0.58	1.91	0.66	1.40
	Social	2.74	0.54	2.68	0.47	2.71	0.51	2.67	0.48	0.17
	Memorization	3.86	0.35	3.77	0.52	3.98	0.42	3.93	0.46	1.72
Post- test	Vocabulary size	24.31	1.28	16.52	2.34	18.64	1.51	14.02	2.05	236.32***
	Metacognition	4.24	0.53	3.52	0.51	3.14	0.90	3.50	0.80	17.87***
	Cognition	3.98	0.75	3.61	1.04	4.07	0.78	3.36	0.82	6.26***
	Social	2.57	0.50	2.61	0.49	2.71	0.51	2.64	0.49	0.61
	Memorization	4.12	0.74	4.00	0.61	3.45	0.50	3.76	0.43	10.75***

*** p < 0.001.

**FIGURE 2:**
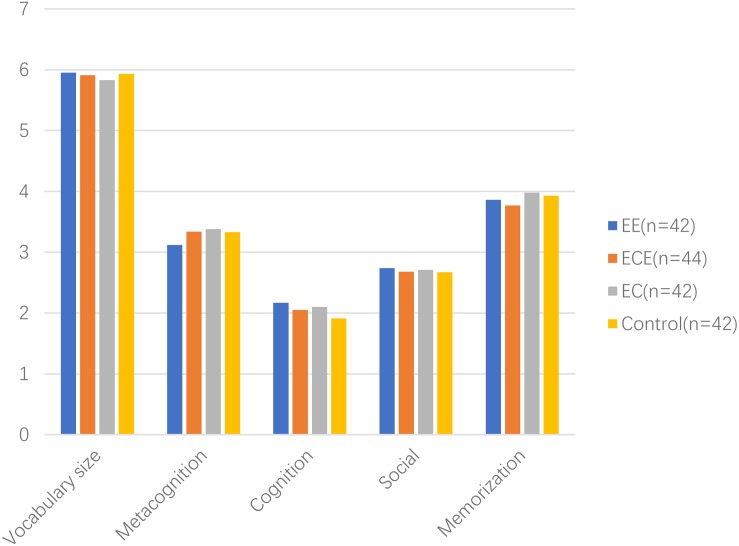
Pre-test results.

**FIGURE 3:**
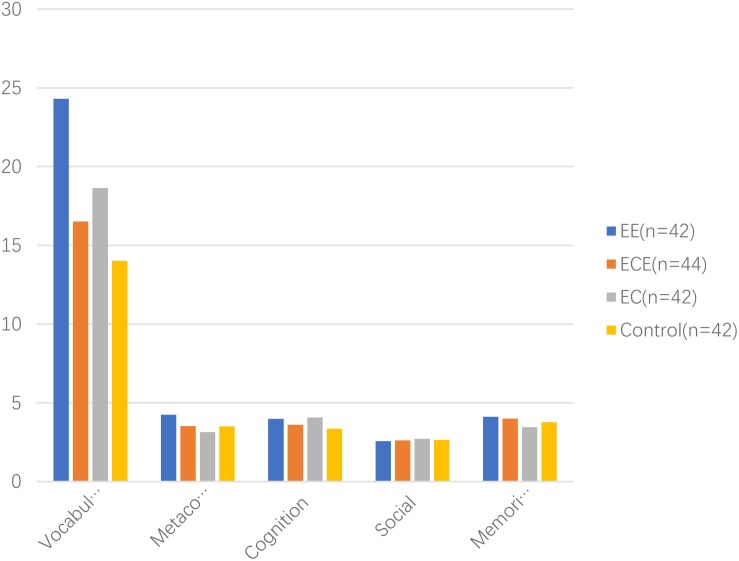
Post-test results.

At post-test, Class 1 (EE) students scored 24.31 (*SD* = 1.28) in receptive vocabulary size test, the meta-cognition score was 4.24 (*SD* = 0.53), the cognition score was 3.98 (*SD* = 0.75), the memorization score was 4.12 (*SD* = 0.74), and the social score was 2.57 (*SD* = 0.50). Class 2 (ECE) students scored 16.52 (*SD* = 2.34) in a receptive vocabulary test, the meta-cognition score was 3.52 (*SD* = 0.51), the cognition score was 3.61 (*SD* = 1.03), the memorization score was 4.00 (*SD* = 0.61), and the social score was 2.61 (*SD* = 0.49). Class 3 (EC) students scored 18.64 (*SD* = 1.51) in receptive vocabulary size test, the meta-cognition score was 3.14 (*SD* = 0.90), the cognition score was 4.07 (*SD* = 0.78), the memorization score was 3.45 (*SD* = 0.50), and the social score was 2.71 (*SD* = 0.51). Class 4 (control group) students scored 14.02 (*SD* = 2.05) in receptive vocabulary size test, the meta-cognition score was 3.50 (*SD* = 0.80), the cognition score was 3.36 (*SD* = 0.82), the memorization score was 3.76 (*SD* = 0.43), and the social score was 2.64 (*SD* = 0.49). All skewness and kurtosis indicators were within ± 3, which showed that the scores were in a normal distribution at post-test. Figures 2, 3 showed the detail information of pre-test and post-test.

### Comparison Analysis

A two-way ANOVA was used to examine the four classes of students’ performance in the receptive vocabulary size test and VLS. Results showed that all four classes of students had a similar score in the receptive vocabulary size test (*F* = 0.17, *p* > 0.10), meta-cognition (*F* = 0.94, *p* > 0.10), cognition (*F* = 1.4, *p* > 0.10), memorization (*F* = 0.17, *p* > 0.10), and social (*F* = 1.72, *p* > 0.10) at pre-test. At post-test, the four classes of students performed significantly differently in the receptive vocabulary size test (*F* = 236.32, *p* < 0.001), meta-cognition (*F* = 17.87, *p* < 0.001), cognition (*F* = 6.26, *p* < 0.001), and memorization (*F* = 10.75, *p* < 0.001). The difference of social score was similar (*F* = 0.62, *p* > 0.10).

A *post hoc* test was further conducted to compare each two classes of students’ performance in the receptive vocabulary size test, meta-cognition, cognition, and memorization. For the receptive vocabulary size test, the difference between Class 1 (EE) and Class 2 (ECE) was significant (mean difference = 7.79, *p* < 0.001), the difference between Class 1 (EE) and Class 3 (EC) was significant (mean difference = 5.67, *p* < 0.001), the difference between Class 1 (EE) and Class 4 (control group) was significant (mean difference = 10.29, *p* < 0.001). The difference between Class 2 (ECE) and Class 3 (EC) was significant (mean difference = -2.12, *p* < 0.001), the difference between Class 2 (ECE) and Class 4 (control group) was significant (mean difference = 2.50, *p* < 0.001). The difference between Class 3 (EC) and Class 4 (control group) was significant (mean difference = 4.62, *p* < 0.001).

For the meta-cognition strategy, the difference between Class 1 (EE) and Class 2 (ECE) was significant (mean difference = 0.72, *p* < 0.001), the difference between Class 1 (EE) and Class 3 (EC) was significant (mean difference = 1.10, *p* < 0.001), the difference between Class 1 (EE) and Class 4 (control group) was significant (mean difference = 0.74, *p* < 0.001). The difference between Class 2 (ECE) and Class 3 (EC) was significant (mean difference = 0.38, *p* < 0.05), the difference between Class 2 (ECE) and Class 4 (control group) was insignificant (mean difference = 0.02, *p* > 0.10). The difference between Class 3 (EC) and Class 4 (control group) was significant (mean difference = -0.36, *p* < 0.05).

For the cognition strategy, the difference between Class 1 (EE) and Class 2 (ECE) was insignificant (mean difference = 0.36, *p* > 0.05), the difference between Class 1 (EE) and Class 3 (EC) was insignificant (mean difference = -0.10, *p* > 0.10), the difference between Class 1 (EE) and Class 4 (control group) was significant (mean difference = 0.62, *p* < 0.01). The difference between Class 2 (ECE) and Class 3 (EC) was significant (mean difference = -0.46, *p* < 0.05), the difference between Class 2 (ECE) and Class 4 (control group) was insignificant (mean difference = 0.26, *p* > 0.10). The difference between Class 3 (EC) and Class 4 (control group) was significant (mean difference = 0.71, *p* < 0.05).

For the memorization strategy, the difference between Class 1 (EE) and Class 2 (ECE) was insignificant (mean difference = 0.12, *p* > 0.10), the difference between Class 1 (EE) and Class 3 (EC) was significant (mean difference = 0.67, *p* < 0.001), the difference between Class 1 (EE) and Class 4 (control group) was significant (mean difference = 0.36, *p* < 0.01). The difference between Class 2 (ECE) and Class 3 (EC) was significant (mean difference = 0.55, *p* < 0.001), the difference between Class 2 (ECE) and Class 4 (control group) was insignificant (mean difference = 0.24, *p* > 0.05). The difference between Class 3 (EC) and Class 4 (control group) was significant (mean difference = -0.31, *p* < 0.05).

The multiple Wilcox test was used to compare the difference in VLS performance between the pre-test and post-test. Results showed that the receptive vocabulary test score was significantly higher in the post-test than in the pre-test for all groups’ students (*W* = 78.21, *p* < 0.001 for EE; *W* = 28.60, *p* < 0.001 for ECE; *W* = 48.63, *p* < 0.001 for EC; *W* = 23.77, *p* < 0.001 for control group). For the meta-cognition strategy, only EE students performed significantly higher in the post-test than in the pre-test (*W* = 7.74, *p* < 0.001), ECE (*W* = 1.30, *p* > 0.10), EC (*W* = 1.24, *p* > 0.10), and control (*W* = 0.98, *p* > 0.10) students performed similarly in both the pre-test and the post-test. For the cognition strategy, all group students performed significantly higher in the post-test than in the pre-test (*W* = 12.05, *p* < 0.001 for EE; *W* = 8.78, *p* < 0.001 for ECE; *W* = 13.23, *p* < 0.001 for EC; *W* = 8.96, *p* < 0.001 for the control group). For the social strategy, EE had a significantly lower score in the post-test than in the pre-test (*W* = 1.97, *p* < 0.05); the other three groups’ students performed similarly between the pre-test and the post-test (*W* = 0.66, *p* > 0.10 for ECE; *W* = 0.01, *p* > 0.10 for EC; *W* = 0.23, *p* > 0.10 for the control group). For the memorization strategy, all groups’ students performed significantly higher in the post-test than in the pre-test (*W* = 2.07, *p* < 0.05 for EE; *W* = 1.88, *p* < 0.05 for ECE; *W* = 5.21, *p* < 0.001 for EC; *W* = 1.98, *p* < 0.05 for the control group).

### Receptive Vocabulary Size Effect Examination

We created three dummy variables of the medium of word explanation (EE, ECE, EC, and control group), and selected the control group as the baseline see Table 2. The partial correlation test between VLS and receptive vocabulary size at the post-test was applied by controlling for the effect of the medium of word explanation. The result showed that the correlation between vocabulary size and each VLS was insignificant (*p* > 0.05), indicating that the vocabulary size did not predict the VLS significantly.

**TABLE 2:** Partial correlation between vocabulary size and vocabulary learning strategies in post-test.

	Meta-cognition	Cognition	Social	Memorization
Post-test	0.05	0.08	0.04	−0.08

## Discussion

The current study had two main findings. First, mediums of word explanation impact Chinese students’ English VLS development significantly. Specifically, EE had a significant positive effect on meta-cognitive strategy, cognitive strategy, and memorization strategy. EE had a significant negative effect on social strategy. ECE and EC had a significant positive effect on cognitive strategy and memorization strategy. ECE and EC had an insignificant effect on meta-cognitive strategy and social strategy. Second, students’ English receptive vocabulary size had an insignificant correlation with VLS.

### Meta-Cognition Strategy

Results showed that the use of metacognitive strategies in the EE medium of word explanation increased significantly. Besides, no significant change is observed in the use of metacognitive strategies in the ECE, EC, and control group students. Different levels of meta-cognitive strategies used in different mediums of word explanation may be related to students’ actual awareness of the gap between their current L2 level and the difficulties of the learning materials (Berger and Karabenick, 2016; Rashid et al., 2016). Thus, it might be possible that exclusive L2 explanation learning materials serve as a signal triggering students’ awareness of their linguistic limitations and the difficulty of the tasks at hand, thereby activating their frequent use of meta-cognitive strategies. Students with a small-size vocabulary might find it hard to understand the exclusive L2-explanation materials (e.g., Shiotsu and Weir, 2007). Being aware that comprehension is not occurring might trigger students to search for alternative methods to comprehend the L2 input. They might try to involve mental manipulation of the target words, such as analyzing, reworking, or associating with already-known knowledge (Craik, 2002).

For EC, a similar reason might be that the provision of cue-L1 translation (Chinese translation) either reduced the difficulties of a task or presented an easy task to the students. Empirical studies showed that Chinese translation was the most effective method for vocabulary learning (e.g., Moskovsky et al., 2015; Tian and Hennebry, 2016). When provided with L1 translation, learners could speed up the process of vocabulary growth (Nation, 2001: 296–316; Ou-Yang and Wu, 2017; Zhang et al., 2017). This may be a result of clear, short, and familiar definitions of L1 translations (Wang, 2015; Carrol et al., 2016). Thus, L1 translation cues were likely to limit students’ L2 meta-cognition strategy development to further evaluate or exercise various strategies in ensuring the occurrence of comprehension (Baumann et al., 1993; Gablasova, 2015).

For ECE, students might find that the learning materials were not too easy or too difficult to learn. Although English explanation materials might hinder students’ comprehension of vocabulary, the L1 translation could serve as a compensatory tool for lexical gaps (e.g., Ong and Zhang, 2010). That is, if students found it difficult to comprehend the L2 explanation, they could opt to check out meanings from L1 explanation. This compensatory effect creates a learning condition that students might have difficulties understanding the L2 explanation but the L1 equivalent could solve this problem. Thus, students’ awareness of their current limitations in the meta-cognition process might not be fully activated, leading to no significant changes in their use of metacognitive strategies.

### Cognition Strategy

The findings observed in all groups’ students with the increased use of cognition strategies are partly consistent with the results of Lawson and Hogben’s (1996) study. Lawson and Hogben (1996) found that students’ use of repetition was related to the limited time given. The observed higher repetition use after over 8 months of learning in our study seems to dispute this factor. There are other three possible reasons for this. First, it might be the over-stimulation of English materials. Since grade 7, systematically, English course teaching was regular in school academic activities. The School-provided English materials might be cognitively loaded with too much distracting information (Gablasova, 2015; Heidari-Shahreza and Tavakoli, 2016). Due to their limited L2 network, students might process the sentence more slowly. Therefore, they might try to cognitively select key information from the L2 explanation, such as taking notes, writing down the keywords, and other strategies. Second, students’ syntactic knowledge might enable them to select the crucial information about word meanings (Omaki and Lidz, 2015; Brimo et al., 2017). It could be assumed that our participants, even with a low English level, were able to process syntactic knowledge, and select crucial elements of the meaning of the words in sentences.

### Social Strategy

EE students had a lower score in social strategies in the post-test than in the pre-test. However, students in the ECE and EC groups showed an insignificant difference in the use of social strategies at the end of the study. The different patterns of using social strategies among EE, EC, and ECE students might be due to students’ cognitive adjustment to adapt to the difficulty of the learning materials. According to the assimilation–accommodation cycles, environmental adaption has two processes: assimilation and accommodation (Abraham and Renner, 1986). The decreased use of social strategies in the EE medium might be a way of students making cognitive adjustments (accommodation) to understand only L2 explanations. That is to say that students, with their current low level of English proficiency, might fail to interpret the L2 explanations (assimilation). They may feel embarrassed when asked to interpret the target words or may need to ask for help, but with the incorrect question description, children had established an impression on vocabulary meaning interpretation, resulting in a lower score of social strategy.

As for the insignificant difference of social strategy in ECE and EC students between the pre-test and the post-test, the reason should be that the presence of L1 translation might serve as a useful tool for learners to assimilate their current level of knowledge with the interpretation of the explanation. This is because the L1 equivalent enables a more direct link between L2 words and their conceptual representation, which facilitates students’ understanding of the target words (Kroll and Stewart, 1994; Yang et al., 2015). It is posited that the Chinese translation might be a compensatory tool for assimilation to occur, which means that this tool enables students to interpret the meaning of the target words. Therefore, students’ efforts to engage in accommodation (e.g., reaching out for other sources) might be limited.

Students in the control group demonstrated an insignificant use of social strategies. This finding is consistent with Schmitt’s (1997) and Asgari and Mustapha’s (2011) research. Students would not have a higher motivation to learn L2 words due to their limited use in early daily conversation and under the limitations of home L2 literacy resources. For example, Asgari and Mustapha (2011) revealed that L2 learners’ reluctance to ask for help was due to the limited available resources for L2 vocabulary acquisition.

### Memorization Strategy

The results revealed that students in all mediums of word explanation demonstrated significant growth in the use of memorization strategies. This shows that, regardless of the types of mediums of word explanation, the use of memorization strategies was promoted. The findings about the increased use of memorization strategies were in contrast with Gu and Johnson’s (1996) findings, but are consistent with the findings of Shi (2006) and Yang and Dai (2012), which indicated that the retest learning methods were replaced by other memorization strategies, such as association, imagery, and mnemonic strategies. As Shi (2006) pointed out, rapid social changes might be a possible reason influencing students’ learning approaches. Jiang and Smith (2009) examined the learning strategies employed by three generations of English language students in China. They found that learner strategy behaviors were significantly influenced by teachers’ teaching approaches. Therefore, the increased use of the memorization strategy might be due to the influence of teachers’ classroom strategies.

### Implications

To develop one or more effective L2 VLS, this study suggested that the online App learning mediums are available to enhance students’ L2 VLS. First, to improve students’ cognitive strategy and memorization strategy, all mediums of word explanation should be available to apply in teaching activities. Second, students’ meta-cognitive strategy could be improved through EE mediums only. However, students would perform worse in the social strategy if teachers applied EE mediums. To meet the requirement of students’ L2 VLS, teachers should select the appropriate mediums of word explanation for teaching activities.

### Limitations

There are several limitations to this study. First, this study confirmed that the learning effect on learners who regarded logographical scripts as the first language, for those learners’ learning effect whose first language is alphabetical scripts, is unknown. Second, self-report data in the responses may run the risk of participant bias in this study (Benson, 2013). When participants were asked to make judgments, they risk drawing from inaccurate sources, such as using self-deception (Paulhus and Vazire, 2007). Furthermore, self-report data may create a gap between the strategies students reported they used and those they actually used. Future research could use qualitative methods, such as a think-aloud protocol, to analyze whether L2 learners know how to use particular VLS.

## Conclusion

Our study revealed that mediums of word explanation have a significant impact on learners’ use of different L2 VLS. To be specific, exposing students to an exclusive L2 medium of word explanation helps enhance their metacognitive awareness and their metacognitive monitoring system. Our study also showed that single word explanations’ scripts might increase their use of VLS better than bilingual word explanations. Moreover, with the provision of the L1 translation provided by teachers at hand, students might lose the tendency to reach out to seek social help. Therefore, L2 educators might need to carefully choose the medium of explanatory materials when designing vocabulary learning materials for their students. It must be noted that our study also argued for the potential effects of teaching approaches of L2 VLS. However, more research needs to be conducted to substantiate this claim.

## Data Availability Statement

The datasets generated for this study are available on request to the corresponding author.

## Ethics Statement

The studies involving human participants were reviewed and approved by the Jiaying University Academic Ethical Committee. Written informed consent to participate in this study was provided by the participants’ legal guardian/next of kin.

## Author Contributions

YD drafted the manuscript. YT did data analysis. SW provided critical comments. W-YD provided the theoretical contribution. ZL provided language editing and comments.

## Conflict of Interest

The authors declare that the research was conducted in the absence of any commercial or financial relationships that could be construed as a potential conflict of interest.
